# Supplementary material for: ProtoMol: enhancing molecular property prediction via prototype-guided multimodal learning
Source: Brief Bioinform. 2025 Dec 8;26(6):bbaf629. doi: 10.1093/bib/bbaf629 (PMC12684735; doi:10.1093/bib/bbaf629)
Supplement: supplement_bbaf629 [file supplement_bbaf629.pdf]

Journal Title Here, 2022, pp. 1–4

doi: DOI HERE
Advance Access Publication Date: Day Month Year
Paper

PAPER

Supplement of ProtoMol

Yingxu Wang*,[1] Kunyu Zhang*,[2] Jiaxin Huang,[1] Nan Yin,[3] Siwei Liu†[4] and Eran Segal†[1,5]

[1]Department of Machine Learning, Mohamed bin Zayed University of Artificial Intelligence, AI Diyafah St, 7909, Abu Dhabi, United Arab Emirates, [2]International College, Zhengzhou University, Daxue North Road, 450000, Henan, China, [3]Department of Computer Science and Engineering, Hong Kong University of Science and Technology , Hong Kong, China, [4]School of Natural & Computing Science, University of Aberdeen, 32 Elphinstone Rd, AB24 3EU, Scotland, United Kingdom and [5]Department of Molecular Cell Biology, Weizmann Institute of Science, Rehovot, Israel

*Equal Contributions.†Corresponding authors: siwei.liu@abdn.ac.uk, Eran.Segal@weizmann.ac.il

FOR PUBLISHER ONLY Received on Date Month Year; revised on Date Month Year; accepted on Date Month Year

Abstract

Key words:

© The Author 2022. Published by Oxford University Press. All rights reserved. For permissions, please e-mail: journals.permissions@oup.com

5. Appendix

5.1 More details about baseline methods

In this part, we introduce the details of the compared baselines as follows:

Textual methods. We compare ProtoMol with two textual methods:

- **SMILES2vec:** [1] is a SMILES-based model that applies deep neural networks over tokenized SMILES sequences, using embedding layers followed by recurrent or convolutional layers to encode sequential chemical structure.
- **SMILES-BERT:** [2] is a SMILES-based model that leverages large-scale unsupervised pre-training using a BERT-style masked language modeling objective, followed by fine-tuning on downstream molecular property prediction tasks.

Graph-based methods. We compare ProtoMol with fourteen graph-based methods:

- **GraphPT:** [3] proposes a general GNN pre-training framework to capture local and global structural information.
- **GraphSAGE:** [4] introduces an inductive framework for learning node embeddings by sampling and aggregating features from a node's local neighborhood, enabling generalization to unseen nodes and efficient learning on large-scale graphs.
- **DGI:** [5] proposes an unsupervised graph representation learning method by maximizing mutual information between local node embeddings and a global summary vector of the graph, using a contrastive objective to distinguish positive samples from corrupted negatives.
- **JOAOv2:** [6] automates the selection of graph augmentations for contrastive learning by jointly optimizing augmentation probabilities and encoder parameters, enabling adaptive and effective pre-training without manual augmentation tuning.
- **GraphCL:** [7] introduces a framework that performs contrastive learning on graphs by applying random graph augmentations and maximizing agreement between augmented views of the same graph to learn expressive and generalizable graph-level representations.
- **GraphLoG:** [8] proposes a self-supervised framework that captures both local and global structural patterns by designing a contrastive objective between instances sampled from the same graph and graphs sampled from the same class, encouraging representations to encode multi-scale topological semantics.
- **MICRO-Graph:** [9] introduces a pre-training strategy that leverages biologically meaningful graph motifs as semantic priors. The method generates motif-aware positive samples and contrasts them against randomly corrupted negatives, enabling the learned representations to emphasize motif-relevant substructures and enhance downstream task performance.
- **MGSSL:** [10] proposes a self-supervised framework that leverages chemical motifs as supervision. It detects frequent substructures in molecular graphs and applies contrastive learning to align motif-level and global representations, enhancing downstream property prediction.
- **GraphFP:** [11] introduces a self-supervised learning framework where molecules are decomposed into fragments. The model is pretrained to predict the presence and arrangement of these fragments, capturing meaningful substructure patterns. During finetuning, the learned fragment-aware representations are transferred to downstream molecular property prediction tasks.
- **GROVE:** [12] proposes a transformer-based architecture pretrained using a combination of contextual property prediction and motif prediction tasks. It leverages graph-level and substructure-level objectives to capture both global and local chemical semantics, enabling effective transfer to molecular property prediction tasks.
- **SimSGT:** [13] introduces a refined masked graph modeling approach that rethinks the design of tokenizers and decoders. It proposes a domain-aware graph tokenizer that generates discrete tokens from molecular graphs and a relation-aware decoder that reconstructs masked graph components, improving molecular representation quality.
- **MoAMa:** [14] introduces a motif-guided masking strategy that incorporates chemical substructure (motif) information into the attribute masking process. By prioritizing the masking of functionally significant atoms and bonds, the method enhances pretraining effectiveness and guides the model to learn chemically meaningful representations.
- **Uni-Mol:** [15] proposes a unified framework for 3D molecular representation learning using SE(3)-equivariant positional encoding and a Transformer backbone. It is pre-trained on large-scale 3D molecular data via masked atom prediction and position denoising, supporting downstream tasks like property prediction and conformation generation.
- **S-CGIB:** [16] introduces a pre-training framework where GNNs learn molecular representations through a subgraph-conditioned Graph Information Bottleneck (GIB). It generates subgraphs as conditionals and trains GNNs to preserve task-relevant information while compressing irrelevant features, improving generalization to downstream molecular tasks.

Multimodal methods. We compare ProtoMol with three multimodal methods that integrate textual and graph-based features:

- **Tri-SGD:** [17] proposes a fusion-based framework that integrates SMILES-based chemical language representations with molecular graph structures. It encodes SMILES using sequence models and graphs using GNNs, and then fuses the two modalities via concatenation followed by dense layers to jointly learn features for drug property prediction.
- **MMSG:** [18] introduces a dual-encoder framework where SMILES strings are processed using Transformer-based encoders and molecular graphs are encoded via GNNs. The model performs modality-specific encoding followed by joint embedding fusion through a shared representation space to enhance molecular property prediction.
- **MDFCL:** [19] proposes a contrastive learning framework that integrates SMILES and graph modalities. It uses dual encoders to extract representations from each modality and employs cross-modal contrastive objectives to align them, enhancing semantic consistency and improving prediction performance.

Algorithm 1 ProtoMol: Enhancing Molecular Property Prediction via Prototype-Guided Multimodal Learning

Require: Molecular graph $\mathcal{G} = (V, E, X)$, textual description $\mathcal{T}$, label y, hyperparameters $(N, K, \lambda_{align}, \lambda_{pred}, \lambda_{proto})$
Ensure: Trained parameters Θ, prediction $\hat{y}$

Initialization: Initialize class-specific prototype set $\mathcal{P} = \{\mathbf{p}_1, \ldots, \mathbf{p}_N\}$ with learnable parameters.
for each training step **do**
  Graph Encoding: $\{\mathbf{z}_g^{(l)}\}_{l=1}^{L} \leftarrow \mathrm{GNN}(\mathcal{G})$
  Text Encoding: $\{\mathbf{z}_t^{(l)}\}_{l=1}^{L} \leftarrow \mathrm{Transformer}(\mathcal{T})$
  for $l = 1, \ldots, L$ **do**
    Cross-Modal Interaction: $(\tilde{\mathbf{z}}_g^{(l)}, \tilde{\mathbf{z}}_t^{(l)}) \leftarrow \mathrm{CrossAttn}(\mathbf{z}_g^{(l)}, \mathbf{z}_t^{(l)})$
    Feature Fusion: $\hat{\mathbf{z}}_g^{(l)} = \mathbf{z}_g^{(l)} + \tilde{\mathbf{z}}_g^{(l)}$, $\hat{\mathbf{z}}_t^{(l)} = \mathbf{z}_t^{(l)} + \tilde{\mathbf{z}}_t^{(l)}$
  end for
  Prototype Projection: Map $\hat{\mathbf{z}}_g^{(L)}$ and $\hat{\mathbf{z}}_t^{(L)}$ into shared space $\mathcal{P}$ to obtain similarity scores α_g, α_t.
  Top-K Selection: Retain the top-K prototypes with highest activations for each sample.
  Loss:

$$\mathcal{L}_{total} = \lambda_{pred}\,\mathcal{L}_{pred} + \lambda_{align}\mathcal{L}_{align} + \lambda_{proto}\,\mathcal{L}_{proto}.$$

  Parameter Update: $\Theta \leftarrow \Theta - \eta \nabla_{\Theta} \mathcal{L}_{total}$
end for
Prediction: Given a new sample, from $\{\,\hat{\mathbf{z}}_g^{(i)}\}_{i=1}^{L}$ to obtain $\hat{\mathbf{o}} = \frac{1}{L}\sum_i \mathrm{Linear}^{(i)}(\hat{\mathbf{z}}_g^{(i)})$, and output

$$\hat{y} = \begin{cases} \arg\max_c \hat{\mathbf{o}}_c, & \text{(classification)} \\ \hat{\mathbf{o}}, & \text{(regression)}. \end{cases}$$

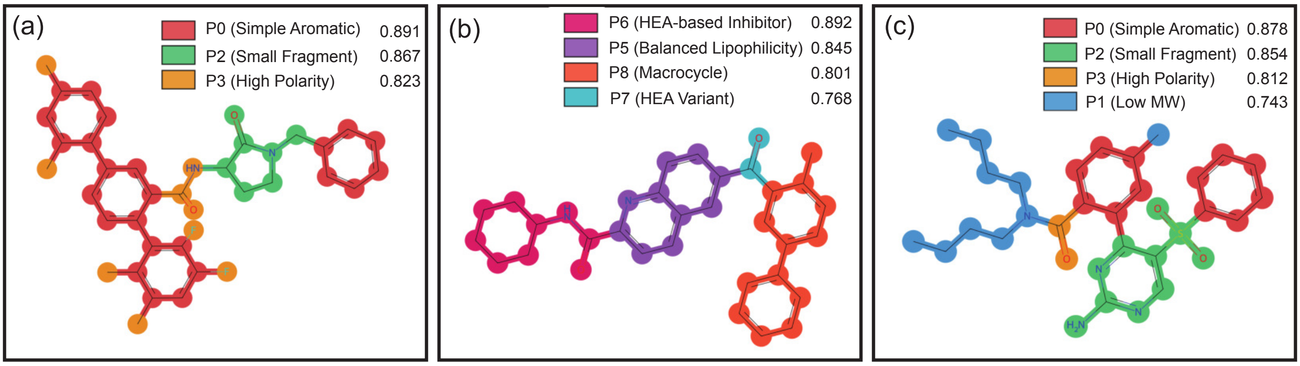

Fig. 1. Visualization of prototype activations for active (b) and inactive (a,c) molecules on the BACE dataset.
Alt text: Visualization of prototype activations for active (b) and inactive (a,c) molecules on the BACE dataset.

5.2 Algorithm

We provide the overall process of ProtoMol in Algorithm 1. ProtoMol. The molecular graph $\mathcal{G}$ is encoded by a multi-layer GNN to capture hierarchical structural representations, while the textual description $\mathcal{T}$ is encoded through a Transformer to extract semantic information. At each layer, bidirectional cross-modal attention enables the interaction between graph and text embeddings, yielding refined representations that jointly preserve molecular topology and contextual semantics. The fused representations are projected into a shared prototype space $\mathcal{P}$, from which the top-K activated prototypes are selected to guide prediction. The model is optimized end-to-end using a composite loss that combines the task-specific predictive loss $\mathcal{L}pred$, the cross-modal alignment loss $\mathcal{L}align$, and the prototype contrastive loss $\mathcal{L}_{proto}$. The final output $\hat{y}$ is obtained by aggregating the layer-wise graph representations and mapping them through the learned prototypes for classification or regression tasks.

5.3 Case-Level Visualization of Prototype Activations

We performe a case-level visualization and analysis of prototype activations to interpret the learned molecular representations more clearly. We trained ProtoMol on the BACE dataset and randomly selected two inactive molecules (in Fig. 1 (a) and (c)) and one active molecule (in Fig. 1 (b)) for prototype-based prediction and visualization. In addition, we quantified the activation strength of each prototype within its corresponding molecule, where higher values indicate stronger prototype responses.

As shown in Fig. 1, distinct prototypes are activated for different molecular classes. For the inactive molecules in Fig. 1 (a) and Fig. 1 (c), the activated prototypes correspond to simple aromatic or polar fragments such as benzene rings, amide linkages, and carbonyl groups, with Prototype P0 exhibiting the strongest activation. In contrast, the active molecule in Fig. 1 (b) primarily activates prototypes associated with more complex and biologically relevant substructures, including cyclohexylamide, naphthalene systems, biphenyl scaffolds, and carbonyl bridges, with Prototype P6 showing the highest activation strength. These observations

demonstrate that prototype activations differ substantially between active and inactive molecules and correspond to chemically meaningful structural motifs, highlighting both the interpretability and chemical significance of ProtoMol's learned prototypes.

References

1. Garrett B. Goh, Nathan Oken Hodas, Charles Siegel, and Abhinav Vishnu. Smiles2vec: An interpretable general-purpose deep neural network for predicting chemical properties. *ArXiv*, abs/1712.02034, 2017.
2. Sheng Wang, Yuzhi Guo, Yuhong Wang, Hongmao Sun, and Junzhou Huang. Smiles-bert: Large scale unsupervised pre-training for molecular property prediction. *Proceedings of the 10th ACM International Conference on Bioinformatics, Computational Biology and Health Informatics*, pages 429–436, 2019.
3. Weihua Hu, Bowen Liu, Joseph Gomes, Marinka Zitnik, Percy Liang, Vijay S. Pande, and Jure Leskovec. Strategies for pre-training graph neural networks. *arXiv: Learning*, 2019.
4. William L. Hamilton, Zhitao Ying, and Jure Leskovec. Inductive representation learning on large graphs. In *Neural Information Processing Systems*, 2017.
5. Petar Veličković, William Fedus, William L Hamilton, Pietro Liò, Yoshua Bengio, and R Devon Hjelm. Deep graph infomax. *arXiv preprint arXiv:1809.10341*, 2018.
6. Yuning You, Tianlong Chen, Yang Shen, and Zhangyang Wang. Graph contrastive learning automated. In *International Conference on Machine Learning*, 2021.
7. Yuning You, Tianlong Chen, Yongduo Sui, Ting Chen, Zhangyang Wang, and Yang Shen. Graph contrastive learning with augmentations. *ArXiv*, abs/2010.13902, 2020.
8. Minghao Xu, Hang Wang, Bingbing Ni, Hongyu Guo, and Jian Tang. Self-supervised graph-level representation learning with local and global structure. In *Proceedings of the 38th International Conference on Machine Learning*, volume 139, pages 11548–11558, 2021.
9. Shichang Zhang, Ziniu Hu, Arjun Subramonian, and Yizhou Sun. Motif-driven contrastive learning of graph representations. *arXiv preprint arXiv:2012.12533*, 2020.
10. Zaixi Zhang, Qi Liu, Hao Wang, Chengqiang Lu, and Chee-Kong Lee. Motif-based graph self-supervised learning for molecular property prediction. *Advances in Neural Information Processing Systems*, 34:15870–15882, 2021.
11. Kha-Dinh Luong and Ambuj K Singh. Fragment-based pretraining and finetuning on molecular graphs. *Advances in Neural Information Processing Systems*, 36:17584–17601, 2023.
12. Yu Rong, Yatao Bian, Tingyang Xu, Weiyang Xie, Ying Wei, Wenbing Huang, and Junzhou Huang. Self-supervised graph transformer on large-scale molecular data. *Advances in neural information processing systems*, 33:12559–12571, 2020.
13. Zhiyuan Liu, Yaorui Shi, An Zhang, Enzhi Zhang, Kenji Kawaguchi, Xiang Wang, and Tat-Seng Chua. Rethinking tokenizer and decoder in masked graph modeling for molecules. *Advances in Neural Information Processing Systems*, 36:25854–25875, 2023.
14. Eric Inae, Gang Liu, and Meng Jiang. Motif-aware attribute masking for molecular graph pre-training. *arXiv preprint arXiv:2309.04589*, 2023.
15. Gengmo Zhou, Zhifeng Gao, Qiankun Ding, Hang Zheng, Hongteng Xu, Zhewei Wei, Linfeng Zhang, and Guolin Ke. Uni-mol: A universal 3d molecular representation learning framework. In *The Eleventh International Conference on Learning Representations*, 2023.
16. Van Thuy Hoang and O-Joun Lee. Pre-training graph neural networks on molecules by using subgraph-conditioned graph information bottleneck. *ArXiv*, abs/2412.15589, 2024.
17. Xiaohua Lu, Liangxu Xie, Lei Xu, Rongzhi Mao, Xiaojun Xu, and Shan Chang. Multimodal fused deep learning for drug property prediction: Integrating chemical language and molecular graph. *Computational and Structural Biotechnology Journal*, 23:1666–1679, 2024.
18. Tianyu Wu, Yang Tang, Qiyu Sun, and Luolin Xiong. Molecular joint representation learning via multi-modal information of smiles and graphs. *IEEE/ACM transactions on computational biology and bioinformatics*, 20(5):3044–3055, 2023.
19. Xu Gong, Maotao Liu, Qun Liu, Yike Guo, and Guoyin Wang. Mdfcl: Multimodal data fusion-based graph contrastive learning framework for molecular property prediction. *Pattern Recognition*, 163:111463, 2025.
